# Cancer incidence and mortality in patients diagnosed with heart failure: results from an updated systematic review and meta-analysis

**DOI:** 10.1186/s40959-023-00158-1

**Published:** 2023-01-25

**Authors:** Massimiliano Camilli, Juan Guido Chiabrando, Marco Lombardi, Marco Giuseppe Del Buono, Rocco Antonio Montone, Antonella Lombardo, Filippo Crea, Giorgio Minotti

**Affiliations:** 1 Department of Cardiovascular and Pulmonary Sciences, Catholic University of the Sacred Heart, 00128 Rome, Italy; 2 Department of Cardiovascular Medicine, Fondazione Policlinico Universitario A. Gemelli IRCCS, 00128 Rome, Italy; 3 Department of Interventional Cardiology, Hospital Italiano de Buenos Aires, Buenos Aires, Argentina; 4 Department of Medicine, Center for Integrated Research and Unit of Drug Sciences, Campus Bio-Medico University and Fondazione Policlinico Universitario Campus Bio-Medico, Rome, Italy

**Keywords:** Heart failure, Cancer incidence, Cancer mortality, Systematic review, Meta-analysis, Cardio-oncology

## Abstract

**Background:**

Several cohort studies aimed at demonstrating an increased risk of cancer incidence and mortality in patients with a pre-existing diagnosis of heart failure (HF); however, conflicting results have been reported that call for systematic review and meta-analysis.

**Methods:**

We conducted a systematic search of multiple databases from their inception through July 2022 and retrieved only papers reporting hazard ratios (HR). Random and fixed-effects models were fit for the study duration.

**Results:**

The analysis included nine cohort studies for a total of 515′041 HF cases and 1′365’452 controls without HF. Although high heterogeneity among studies was observed, the HR for incident cancer in HF patients was statistically significant (1.45, 95% CI 1.31–1.61, *p < 0.0001*), which was confirmed by sensitivity analyses; however, by analyzing the few papers reporting HRs for cancer mortality, no significant difference between HF and non-HF patients could be detected (HR 2.03, 95% CI [0.93–4.43], *p = 0.0736*). Further scrutiny of studies with adjusted HRs, when available, confirmed that cancer incidence was significantly increased in patients with HF, as was cancer mortality as well.

**Conclusions:**

This meta-analysis shows that HF patients are at an increased risk of incident cancer. Increased mortality could not be firmly demonstrated by the available data. Our results call for inclusion of cancer-related endpoints in HF trials to adequately address this important clinical issue.

## Background

With progressive aging of the general population, there is an increasing intersection of cardiovascular diseases (CVDs) with cancer, raising questions around possible cause-and-effect relations between these two diseases [1–3]. Heart failure (HF) is one of the most severe complications of many CVDs, and over the past decades we witnessed an exponential growth in its incidence [4].

Cancer patients may develop HF due to the well-known cardiotoxic effects of antineoplastic treatments [5]. Therefore, in order to provide better care to subjects experiencing cardiotoxicity, cardio-oncology was born and gained a relevant role in cardiology settings [5]. Whereas HF risk from cancer drugs has been actively investigated in numerous clinical trials, only few data are available in the setting of “reverse cardio-oncology”, which looks at cancer incidence in patients with pre-existing HF [6]. As a matter of fact, patients with HF are usually excluded from oncological clinical trials because of concerns about cardiotoxicity of cancer drugs, the high prevalence of comorbidities and worse prognosis compared to the general population [1–3]. Oncological trials cannot therefore tell us how many patients were affected by HF before cancer was diagnosed.

Over the last years there have been analyses of community-based cohorts suggesting a higher prevalence of cancer in subjects with heart failure (HF) compared with those without HF, even when the influence of shared risk factors, surveillance biases and cardiovascular medications were taken in due consideration [1–3]. The association of cancer with HF may have important implications [1–3, 6], fostering research on common pathophysiological mechanisms and setting the stage for preventive cancer screening in HF patients and identification of new targets for pharmacological interventions. However, the link between HF and cancer was not confirmed in all available studies [7]. We therefore aimed at performing a systematic review and meta-analysis of cancer incidence and mortality in patients diagnosed with HF.

## Methods

This systematic review and meta-analysis was in accordance with the Preferred Reporting Items for Systematic Reviews and Meta-analysis (PRISMA). We conducted a systematic search of PubMed, Google Scholar, Embase and reference lists of relevant articles, from their inception through July 2022. To identify all potentially relevant papers a combination of the following terms was used: “heart failure”, “cancer or tumor or malignancy”, “incidence”, “mortality”. Studies deemed relevant to our search were downloaded and the full manuscripts reviewed. Studies were eligible for inclusion if they were aimed at evaluating cancer incidence in patients with a pre-existing diagnosis of HF and reported hazard ratios (HRs). Analyses including adjusted and unadjusted HRs were used to evaluate the incidence of primary and secondary outcomes of interest (cancer incidence and mortality, respectively). Both adjusted and unadjusted ratios were considered. Three investigators (MC, ML, JGC) independently reviewed study titles and abstracts. I^2^ testing was performed to evaluate the magnitude of heterogeneity between studies, which was considered substantial when > 50%. Fixed-effects model was used for low heterogeneity among studies (I^2^ < 50%), while random-effects model was used for high heterogeneity (I^2^ > 50%). Publication bias for small study effect appraisal was assessed by Egger test and Funnel plots. Computations were performed with the R statistical software (4.0.0 version) using “meta” package.

## Results

**Fig. 1:**
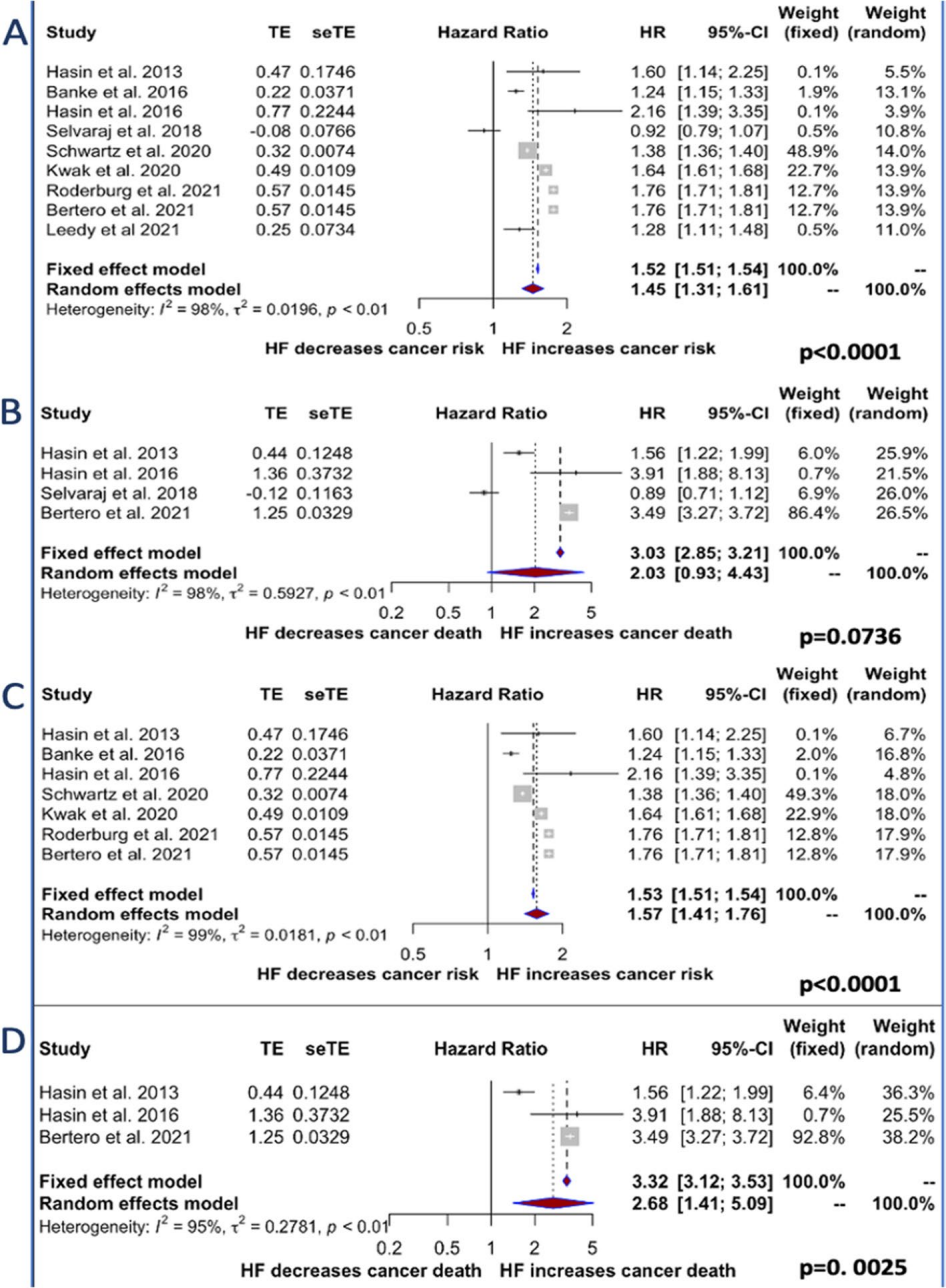
Forest plots for the incidence of the primary and secondary outcomes. Panel **A** cancer incidence in patients with and without Heart Failure (HF). Panel **B** cancer mortality according to previous diagnosis of HF. Panel **C-D** sensitivity analysis evaluating cancer incidence and mortality excluding papers with gender restriction

Of the 1′213 papers examined, 9 met our research criteria and were included in the final analysis, providing data on 515′041 HF cases and 1′365’452 controls without HF [8–16]. Study characteristics are reported in Table 1. In the HR analysis, HF patients had a significantly increased cancer incidence compared to controls without HF (HR 1.45, 95% CI (1.31–1.61), *p < 0.0001*) (Fig. 1, Panel A). However, when papers reporting HRs for cancer mortality were analyzed, no significant difference between the two groups was observed (HR 2.03, 95% CI [0.93–4.43], *p = 0.0736*) (Fig. 1, Panel B). Having considered that two reports included in our analyses had recruited only male or female patients [11, 15], a sensitivity analysis that excluded these papers was conducted and shown to confirm a higher incidence of cancer in subjects with HF (HR 1.57, 95% CI [1.41.1.76], *p < 0.0001*) (Fig. 1, Panel C). The same analysis revealed a statistically significant increase also in mortality (HR 2.6824, 95% CI [1.41–5.08], *p = 0.0025*) (Fig. 1, Panel D).

**Table 1:** Main characteristics of the analyzed studies

***Study***	***Design***	***Total N of Patients***	***Follow-Up Time***	***Exclusion Criteria***	***HF Patients’ Age (years)***	***No HF Patients’ Age (years)***	***Male Sex*** ***HF No HF***		***HF Definition***
***Hasin*** **et al.*, 2013*** [8]	Case-control and prospective cohort study	1.922 (961/961) case-control; 1.192 (596/596) cohort.	7.7 ± 6.4 years	Prior cancer	73,0 ± 14,0	73,0 ± 14,0	47%	47%	Medical chart review (Framingham criteria)
***Banke*** **et al.*, 2016*** [9]	Cohort study	4.968,582 (9304/4.959.275)	4.5 ± 2.3 years	Prior cancer	51,6 ± 7,1	N.A.	72.6%		Clinical evaluation and echocardiography
***Hasin*** **et al.*, 2016*** [10]	Prospective cohort study	1.081 (228/853)	4.9 ± 3 years	Prior cancer, nonmelanoma skin cancer	62,0 ± 15,0	72,0 ± 14,0	46%	63%	Medical chart review (Framingham criteria)
***Selvaraj*** **et al.*, 2018*** [11]	Prospective cohort study (post-hoc analysis of RCT)	28.341 (1.420/26.921)	19.9 years	Prior cancer	61,0 ± 9,0	55,0 ± 1,0	100%		Self report validated by Framingham criteria
***Schwartz*** **et al.*, 2020*** [12]	Retrospective cohort Study	1.004.759 (167.633/837.126)	Mean 3.0 years for HF group/6.8 yeas for control group	Prior cancer	70,9 ± 13,3	70,9 ± 13,3	55%	55%	ICD-10 codes
***Kwak*** **et al.*, 2020*** [13]	Retrospective cohort study	770.646 (128.441/642.205)	4.06 years	Prior cancer	67,1 ± 12,4	67,1 ± 12,4	51.9%	51.9%	ICD-10 code with at least one hospital admission attributed to HF diagnosis
***Roderburg*** **et al.*, 2021*** [14]	Retrospective cohort study	200.248 (100.124/100.124)	10 years	Prior cancer	72,6 ± 12,2	72,6 ± 12,2	46%	46%	ICD-10 code and drug prescriptions
***Leedy*** **et al.*, 2021*** [15]	Post-hoc analysis of a prospective cohort study	146.817 (3.272/104.020)	8.4 years	Prior cancer, no follow-up data, self-reported HF	63,1 ± 6,7	N.A.	None		Clinical review of hospitalization records with primary diagnosis of HF event
***Bertero*** **et al.*, 2021*** [16]	Retrospective cohort study	208.040 (104.020/104.020)	Mean 5.6 years for HF group/5.3 years for controls	Prior cancer; age < 50 years	76,0 ± 10,0	76,0 ± 10,0	46.8%	46.8%	Health Care records on drug prescriptions, outpatients visit reports, health care cost-related fee waivers, death certificates.

**Fig. 2:**
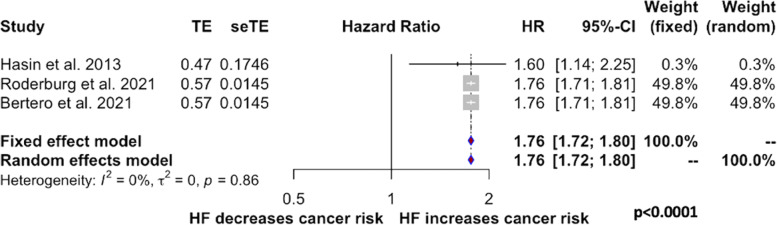
Forest plots for the incidence of the primary outcome (Panel **A**) in analyses including only papers with matched 1:1 case-control design

Heterogeneity among studies was high (incidence: *I*^*2*^ = 98%, *p < 0.01;* mortality: *I*^*2*^ = 95%, *p < 0.01*). This was probably caused by differences in sample size and unbalanced case:control ratios across studies. Therefore, papers with matched 1:1 case:control design were analyzed in isolation. In this secondary analysis there was no detectable heterogeneity among studies and patients with HF showed a significantly higher incidence of cancer (HR 1.76, 95% CI (1.72–1.80), *p < 0.0001*) (Fig. 2). Cancer mortality was not explored because only two studies remained available for this analysis.

**Fig. 3:**
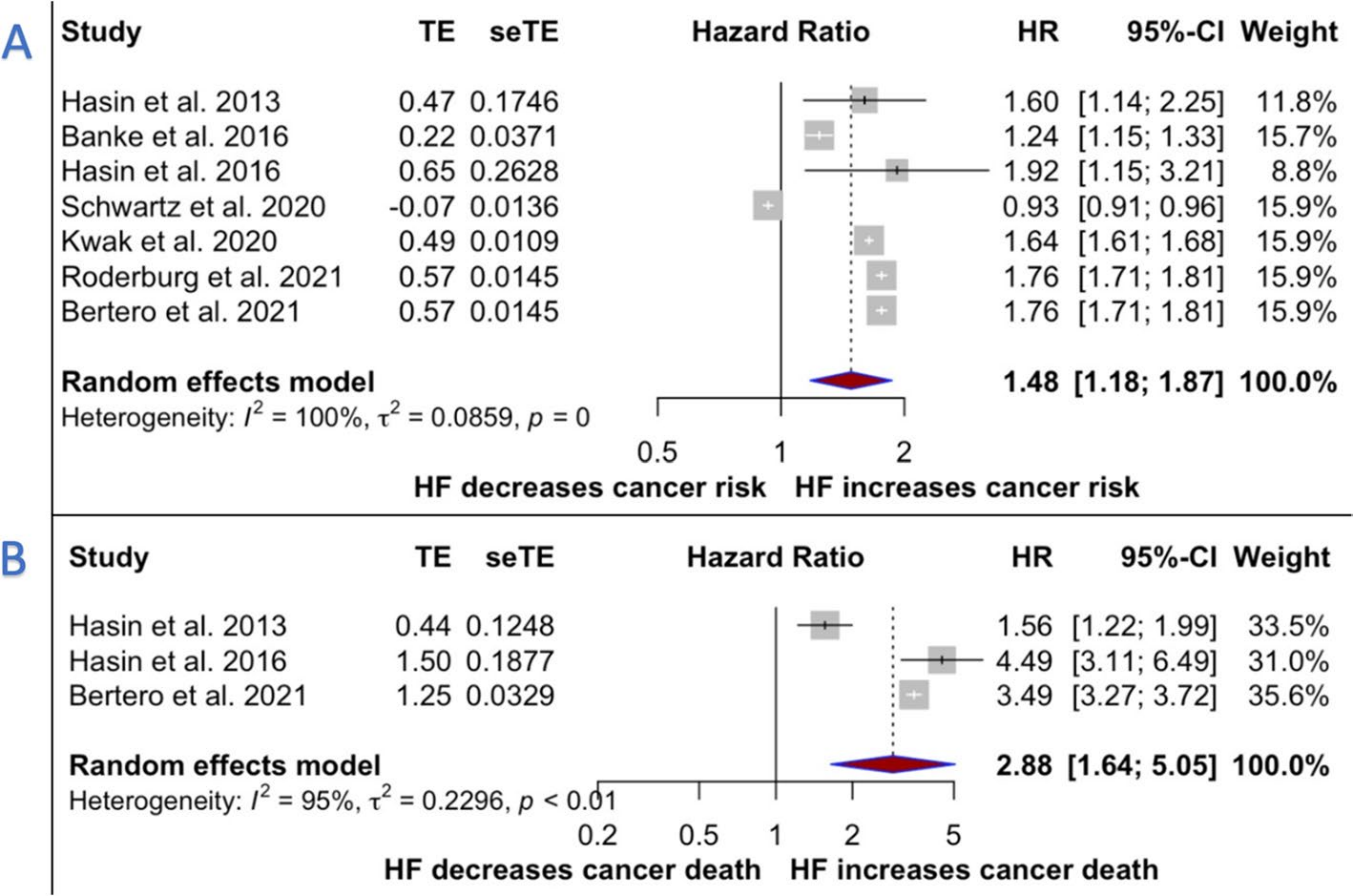
Forest plots for the incidence of the primary and secondary outcomes using adjusted Hazard Ratios. Panel **A** cancer incidence in patients with and without Heart Failure (HF). Panel **B** cancer mortality according to previous diagnosis of HF

Further scrutiny of studies with adjusted HRs (8–10;12–15) confirmed that cancer incidence was significantly increased in patients with HF (HR 1.48, 95% CI [1.18–1.87], *p = 0.0008),* as was cancer mortality as well [8, 10, 15] (HR 2.88, 95% CI [1.64–5.05], *p = 0.0002*). In both analyses, however, heterogeneity was high (Fig. 3, Panel A-B).

## Discussion

To our knowledge, this is the most comprehensive meta-analysis of cancer incidence and mortality in patients diagnosed with HF. As many as nine cohorts were in fact investigated, and cancer incidence proved to be significantly higher in HF patients both in the primary analysis and in the secondary analysis that was performed to reduce heterogeneity among studies. These results extend those reported in another meta-analysis [17] that included only four studies [8–11].

There are solid pathophysiological foundations to explain the higher risk of cancer in HF patients. Preclinical data show that HF generates a pro-neoplastic milieu characterized by secretion of oncogenic factors and neuro-hormonal activation, which in turn promotes tumor development [1–3]. Moreover, HF is characterized by a low-grade systemic inflammation that predisposes to neoplastic transformation and progression [18]. Cancer and HF also share risk factors, such as advanced age, metabolic disorders and smoking habit [19]. Increased cancer risk could well be caused by one or more comorbidities usually observed in patients with HF. This is a rather complex scenario if one considers that e.g., diabetes, obesity, respiratory and renal diseases are almost universally observed in patients affected by HF with preserved ejection fraction [19]. Moreover, HF was shown to associate with enhanced tumor growth, which may be independent of hemodynamic impairment but probably is caused by factors of cardiac origin (e.g. serpinA3) [20].

In our report, cancer mortality in patients with or without HF could be investigated only in the primary analysis, in which differences between the two groups could not be confirmed. For the sake of clarity, we further evaluated cancer mortality when adjusted HRs were available, showing a significantly increased cancer mortality in the HF population compared to controls. The paucity of studies reporting HRs for cancer mortality does nonetheless introduce statistical constraints and precludes firm conclusions.

An overview of clinical trials performed over a timespan of 30 years shows that only 25% of them provided information on cancer mortality in HF patients, mainly because cancer was a protocol-prespecified exclusion criterion. More recently, a meta-analysis of 15 phase 3 clinical trials of HF patients showed that up to 67% of non-cardiovascular deaths were attributable to cancer [21], with the proportion of cancer-related deaths ranging from 6 to 7% and peaking at over 14% of all deaths [21]. Limited number of studies and high heterogeneity between them made our analyses insufficiently powered to detect an increased cancer-related mortality in HF patients. Moreover, active surveillance for HF may have resulted in earlier diagnosis and treatment of cancer, thus modifying mortality rates in HF patients.

Most of the reports included in our analyses were performed with administrative data. This approach presents both strengths and limitations for research purposes. Administrative datasets provide information about large or very large cohorts but this comes at the cost of unmeasured confounders and consequent biases. We therefore acknowledge that our results should be interpreted with due caution. Study limitations should nonetheless be weighed against the fact that our analyses included the most updated sources of cancer incidence in patients with HF.

## Conclusions

Our report shows that HF syndrome portends a higher incidence of cancer, even when secondary analyses eliminated biases from heterogeneity among studies. These results may have implications for research developments and patients’ management. Differences in cancer mortality between HF and non-HF patients cannot be firmly established at this point in time, due to the paucity of studies with HRs for cancer-related death. These facts strongly support the inclusion of oncologic endpoints in clinical trials of HF.

## Data Availability

The datasets used and analysed in this study are available from the corresponding author on reasonable request.

## Abbreviations

CVD: Cardiovascular diseases
HF: Heart failure
HR: Hazard ratio
PRISMA: Preferred Reporting Items for Systematic Reviews and Meta-analysis

## References

[1] de Boer RA, Meijers WC, van der Meer P, van Veldhuisen DJ (2019). Cancer and heart disease: associations and relations. Eur J Heart Fail.

[2] de Boer RA, Hulot JS, Tocchetti CG, Aboumsallem JP, Ameri P, Anker SD (2020). Common mechanistic pathways in cancer and heart failure. A scientific roadmap on behalf of the translational research Committee of the Heart Failure Association (HFA) of the European Society of Cardiology (ESC). Eur J Heart Fail.

[3] Tocchetti CG, Ameri P, de Boer RA, D'Alessandra Y, Russo M, Sorriento D (2020). Cardiac dysfunction in cancer patients: beyond direct cardiomyocyte damage of anticancer drugs: novel cardio-oncology insights from the joint 2019 meeting of the ESC working groups of myocardial function and cellular biology of the heart. Cardiovasc Res.

[4] Adamo M, Gardner RS, McDonagh TA, Metra M (2022). The ‘Ten Commandments’ of the 2021 ESC guidelines for the diagnosis and treatment of acute and chronic heart failure. Eur Heart J.

[5] Lyon AR, López-Fernández T, Couch LS, Asteggiano R, Aznar MC, Bergler-Klein J (2022). 2022 ESC guidelines on cardio-oncology developed in collaboration with the European Hematology Association (EHA), the European Society for Therapeutic Radiology and Oncology (ESTRO) and the international cardio-oncology society (IC-OS). Eur Heart J.

[6] Aboumsallem JP, Moslehi J, de Boer RA (2020). Reverse cardio-oncology: Cancer development in patients with cardiovascular disease. J Am Heart Assoc.

[7] Deswal A, Basra SS (2013). Incident cancer in patients with heart failure: causation or mere association?. J Am Coll Cardiol.

[8] Hasin T, Gerber Y, McNallan SM (2013). Patients with heart failure have an increased risk of incident cancer. J Am Coll Cardiol.

[9] Banke A, Schou M, Videbaek L (2016). Incidence of cancer in patients with chronic heart failure: a long-term follow-up study. Eur J Heart Fail.

[10] Hasin T, Gerber Y, Weston SA (2016). Heart failure after myocardial infarction is associated with increased risk of cancer. J Am Coll Cardiol.

[11] Selvaraj S, Bhatt DL, Claggett B (2018). Lack of association between heart failure and incident cancer. J Am Coll Cardiol.

[12] Schwartz B, Schou M, Gislason GH, Køber L, Torp-Pedersen C, Andersson C (2020). Prevalence and incidence of various Cancer subtypes in patients with heart failure vs matched controls. Int J Cardiol.

[13] Kwak S, Kwon S, Lee SY (2021). Differential risk of incident cancer in patients with heart failure: a nationwide population-based cohort study. J Cardiol.

[14] Roderburg C, Loosen SH, Jahn JK (2021). Heart failure is associated with an increased incidence of cancer diagnoses. ESC Heart Fail.

[15] Leedy DJ, Reding KW, Vasbinder AL (2021). The association between heart failure and incident cancer in women: an analysis of the Women’s health initiative. Eur J Heart Fail.

[16] Bertero E, Robusto F, Rulli E (2022). Cancer incidence and mortality according to pre-existing heart failure in a community-based cohort. J Am Coll Cardiol CardioOnc.

[17] Zhang H, Gao Y, Wang L (2020). Does heart failure increase the risk of incident cancer? A meta-analysis and systematic review. Heart Fail Rev.

[18] Libby P (2006). Inflammation and cardiovascular disease mechanisms. Am J Clin Nutr.

[19] Koene RJ, Prizment AE, Blaes A, Konety SH (2016). Shared risk factors in cardiovascular disease and Cancer. Circulation..

[20] Meijers WC, Maglione M, Bakker SJL, Oberhuber R, Kieneker LM, de Jong S, Haubner BJ, Nagengast WB, Lyon AR, van der Vegt B, van Veldhuisen DJ, Westenbrink BD, van der Meer P, Silljé HHW, de Boer RA (2018). Heart failure stimulates tumor growth by circulating factors. Circulation..

[21] Tini G, Bertero E, Signori A, Sormani MP, Maack C, De Boer RA, Canepa M, Ameri P (2020). Cancer mortality in trials of heart failure with reduced ejection fraction: a systematic review and Meta-analysis. J Am Heart Assoc.

